# Everybody's Page

**Published:** 1890-11-15

**Authors:** 


### Everybody's Page.

The Congress of Prussian Midwives at Berlin seems to have been a great success. The lectures delivered at it are to be published.

Our cousins on the other side of the duck-pond are nothing unless they are immense. But is not their ill-concealed disappointment at the result of the census the reverse of immense?

Some of the possibilities for crime offered by the practice of mesmerism, or hypnotism, as it is now the fashion to call it, were shown recently in Paris, when a hypnotist secured a cheque for 10,000 francs from the victim. The existence of such possibilities has been recognised in a practical manner by the Russian medical department. Their medical council has resolved that henceforward the application of hypnotism for medical purposes can be permitted solely to medical practitioners, under the condition that the operation is to be practised invariably in the presence of other medical men.

If it were generally known that the authorities of any hospital in this country neglected to label properly the medicines administered therein, and allowed poisons intended for external use only to be kept in the same cupboard with medicines for internal use, placing them them under the control of inexperienced probationers, the hospital would very soon have to close its doors. A case of such gross neglect and want of proper supervision is reported by the *Journal of the American Medical Association* to have occurred in Belle Vue Hospital, New York, with disastrous results. The unfortunate patient, who was the victim of this carelessness, lost his life in consequence.

The gold "loof" of Annam, which is worth about £65 sterling, is said to be the largest gold coin in circulation in the world. It would be rather weighty and unwieldy for the modern pocket, but we could put up with such a trifling discomfort.

The recent outbreak of cholera in Spain has been attributed by one French authority to the exhuming of the bodies of the victims of the epidemic of 1885. If this, as is by no means improbable, is the case, yet another cogent reason for cremation is given us. Nothing could point more clearly to the necessity of cremation in cases of deaths from infectious diseases.

It is announced that the School Board intend only to introduce pianos into those schools which have central halls for the purpose of giving an accompaniment of music only to the drill and not of teaching the children to play. Ratepayers will be thankful that their reasonable protests have had some effect. The School Board can advantageously take to heart the case of Patty Patterson, who was recently charged at Southwark with "unlawfully using certain subtle means, and craft, or device—to wit, by cards—to deceive and impose on Her Majesty's subjects." The School Board has quite enough to do in eradicating crass ignorance and superstition without flaunting before the long-suffering ratepayers a flashy and meretricious programme of education. The victims of Mistress Patty Patterson were as usual young girls, mostly from the neighbouring factories. She was described in the charge sheet as of "no occupation," but she appears to have had a thriving and lucrative one. Mistress Patterson received "six weeks"; not many generations ago the wretched woman would have been burnt.

